# Diseases of the Rectum

**Published:** 1838-07-01

**Authors:** 


### Medico-Chirurgical Review,

### No. LVII.

**[No. 17 of a Decennial Series.]**

APRIL 1, 1838, TO JULY 1, 1838.

### DISEASES OF THE RECTUM.

I. A TREATISE ON THE MALFORMATIONS, INJURIES, AND DISEASES OF THE RECTUM AND ANUS. By *George Bushe*, M.D., Formerly Professor of Anatomy and Physiology, &c. New York, 1837. 8vo., pp. 299.

II. ON THE DISEASES OF THE RECTUM. By *James Syme*, F.R.S.E., Professor of Clinical Surgery in the University of Edinburgh. 8vo., pp. 138. Edinburgh, 1838.

WE have occupied two copious articles in the two numbers of this Journal, immediately antecedent to the present, with the important subject of Diseases of the Rectum. The majority of those diseases have been amply treated of, and we intend to dispose of the remainder now.

The principal complaints which require notice are abscess near the anus, and its consequence, fistula in ano—stricture of the rectum—polypi of the rectum—and malignant disease of the gut.

### a. ABSCESS NEAR THE ANUS, AND FISTULA IN ANO.

1. These affections, which frequently stand in the relation of cause and effect, form the subject of two chapters in the work of Dr. Bushe, and of one in that of Mr. Syme. As the former gentleman dwells very circumstantially on abscess near the rectum, and as this is usually the initiative in the production of fistula, we shall principally draw from his pages what we have to say respecting it.

It is not surprising, that the cellular membrane near the anus should be liable to suppuration. The abscesses to which it is prone are divided by Dr. Bushe into those which are independent of disease of the rectum, and those which are produced by a morbid condition of the bowel.

#### *a. Abscesses independent of Disease of the Rectum.*

Dr. Bushe divides these into the idiopathic, traumatic, critical, and symptomatic.

*Idiopathic Abscesses* are either phlegmonous or gangrenous. The phlegmonous may be either acute or subacute.

The acute are ushered in with fever, pain, throbbing, swelling, and induration of the parts in the neighbourhood of the anus, with frequent and difficult micturition. In a few days matter forms, and is discharged either into the intestine or externally, by one or more openings, after which, the pain and fever subside.

The subacute are attended with irritative fever. Generally, they are large, deep-seated, and accompanied with a sense of weight, occasional throbbing, and spasm of the sphincter. The tumefaction, though not very great externally, is, however, very perceptible to the finger *in ano*. The urinary organs sympathize, but not to the same extent as in the acute form of disease just described. These abscesses are slow to burst, and if artificial means be not used, the patient will become exceedingly low, and the rectum will be stripped of its cellular tissue. Sometimes, they open into the rectum, but more commonly, the superincumbent integuments give way in one or more points, and the matter is thus discharged.

Fatigue, deterioration of health, and insufficient nourishment, dispose to this form of disease, and in some instances, seem sufficient for its production; while contusions, sitting on wet seats, and riding on horseback, excite alike the formation both of this and the acute form of abscess.

The gangrenous, like the phlegmonous abscesses, are sometimes acute and sometimes chronic in their character.

The acute generally occur in bad constitutions, especially in those who have lived luxuriously, and are advanced in life. They are preceded by rigors, and attended with fever. In the commencement this is of a more active, subsequently of a typhoid character.

The patient first complains of deep-seated pain by the side of the anus, where we may easily detect a hard point, which soon spreads; then, the pain assumes a burning character, there is considerable tenesmus, and the dysuria is more severe, than in any of the other forms of abscess. The swelling becomes diffused, the tension increases, though not to a very considerable extent, and the skin turns livid. If the diseased parts be laid open, the cellular tissue will be found extensively gangrenous, and infiltrated with very bad pus. Partial openings arise from the mortification of the integuments, and the pus, with portions of cellular tissue, are discharged very slowly. Sometimes, however, the skin and cellular membrane are much more extensively diseased.

The gangrenous abscesses of more chronic character come on almost imperceptibly, the cellular tissue and skin are less extensively diseased, and they are not attended with much fever, or local suffering. Dr. Bushe relates the case of a man, aged 59, who, in October, 1837, began to feel some uneasiness in the anal region near the right ischium, attended with slight swelling, which increased gradually, and assumed a livid aspect. In the following July, when he was seen by Dr. Bushe, the tumefaction was moderate, and without much tension, the integuments were of a dark colour, and there existed two openings, which discharged a small quantity of ill-conditioned pus. His health was, as it had been for years, indifferent. Dr. Bushe laid the diseased parts freely open, and found that the cellular tissue was partly condensed, partly gangrenous, and the seat of two cavities, each about the size of a filbert, with which the openings above mentioned communicated.

*Traumatic Abscesses* are necessarily rare. M. Ribes relates the case of a lieutenant, who received a musket-ball in the centre of the right buttock, which fractured the tuber ischii, and passed into the rectum, as proved by the immediate flow of blood from the anus, and the exit of the ball on the 16th day, by the same outlet. The external wound suppurated freely, and in six weeks had healed; but then, the right side of the perineum inflamed, and seemed, from its bluish appearance, about to become gangrenous, so as to lead Ribes to suspect a stercoraceous abscess; however, he punctured it, but could not detect any opening in the rectum, on the contrary, he found that the walls of this bowel were much thickened. In a few days, he extracted a fragment of bone, and some pieces of cloth, after which the abscess healed, and the patient was restored to health. Dr. Bushe once saw a case not unlike this, in a soldier who was wounded in India.

*Critical Abscesses* occur after fevers and repelled eruptions. Dr. Bushe says little about them. This is hardly the place for their description.

*Symptomatic Abscesses* are divided by Dr. Bushe into two classes, viz. those which form in other organs, and extend downwards by the side of the rectum, and those which arise in connexion with affections of the respiratory organs.

"The first class, includes spinal, urinary, and uterine abscesses. The purulent collections, depending upon disease of the spine, or the soft parts in its neighbourhood, are preceded by symptoms of disease of this column, or these parts; and the matter, after working its way downwards, between the folds of the mesorectum, appears at the verge of the anus, without discolouration, pain, or induration of the surrounding tissues. The urinary abscesses are either gangrenous, arising from the extravasation of urine, or phlegmonous, the urine being prevented from escaping by an organized lymphatic barrier, and are preceded either by disease or injury of the urethra, which easily points out their true character. The abscesses, which arise from disease of the uterus, take place, most commonly, in the advanced stages of cancer of this organ, and discharge a sanious fluid, well marking the malignant character of this fatal disease. The second class occurs in those who labour under chronic laryngeal, tracheal, but more especially, pulmonary disease, and may be accounted for; firstly, by the constant impulse communicated to the anal region by coughing; secondly, by the unsupported condition of the veins in this situation, in the latter stages of consumption, in consequence of the absorption of fat; and thirdly, by the retardation of the portal circulation, which depends upon the pulmonary obstruction; for, as explained in page 17, a free communication exists between the veins of the cellular tissue, without the lower extremity of the rectum, and the hemorrhoidal plexus." 237.

It is rather singular, that the connexion between pulmonary disease and abscess or fistula ani, appears to Mr. Syme to be highly mysterious.

"It is difficult," he says, "to trace the connexion between pulmonary complaints and *fistula in ano*; but no point in pathology is better established than that there is such a connexion; and attention is not unfrequently first drawn to the phthisical condition of a patient by the disposition that he shows to suffer from the disease in question; whence it has sometimes been erroneously supposed that the discharge of the fistula brings on the disease of the lungs. As the great intestine is generally found ulcerated in the bodies of those who have died from consumption, it seems probable that the morbid state of this part, and not that of the lungs, is the exciting cause of fistula, but the disease certainly does occur in cases of pectoral affection, which exhibit no symptom of intestinal disorder." 10.

We cannot perceive the difficulty which appears so insuperable to Mr. Syme. Tubercular depositions in the lungs must interfere in a greater or a less degree with the return of blood from the veins. The cellular membrane in the vicinity of the anus has many veins—these have little adventitious support—the cellular membrane itself is of great depth—the functions of the rectum expose it to a good deal of mechanical disturbance—and these circumstances, taken in connexion with what we know of the properties of the cellular membrane, and of its proneness to suppuration, explain, we conceive, the frequent concurrence of the latter with pulmonic alterations.

*b. Abscesses which arise from a Morbid Condition of the Rectum.*

These are arranged by Dr. Bushe under the heads of idiopathic, traumatic, and symptomatic.

The *Idiopathic* abscesses may arise from three causes; first, the accumulation of fæces, by which the circulation is retarded, and the rectum engorged with blood; secondly, the entanglement of small particles of indurated fæces in the lucunæ; and thirdly, from the slow inflammation which takes place, either in the vicinity or substance of tumefied hæmorrhoidal tumors. From any of these causes, ulceration may ensue, fæcal matter extravasate, and an abscess is the consequence.

The *Traumatic Abscesses* proceed from the passage of balls, punctures, badly directed incisions in lithotomy, and foreign bodies which have been entangled by the internal sphincter. Dr. Bushe collects in a note, some remarkable cases which have been at different times put on record.

"I have seen but one case, in which a stercoraceous abscess resulted from the presence of a foreign body, and this was in the person of a boy, eleven years old, who swallowed, between three and four months previously, a portion of the thigh-bone of a chicken, about half an inch long. He had suffered severely for some weeks before I was called; but the nature of his complaint was not suspected. I laid the parts freely open, and extracted the bone.

There are several interesting cases of this kind on record, of which the following are the most remarkable: Le Dran relates a case, which occurred to M. Destendau, of a man who for nine months laboured under a fistula caused by the lodgment of a piece of bone. (Observations de Chirurgie, tome second. Observation lxxxvi. p. 222. Paris, M,DCC,XXXI.) Petit mentions some cases of this kind. In one, he extracted a needle, which, for six months, had given rise to excruciating pain during defecation. In a second, he removed a small triangular bone, which for four or five months, had created great suffering. In a third there was extensive mortification of the parts surrounding the anus, in consequence of the lodgment of a chicken bone, of ten years duration. Finally, in a fourth he opened an abscess, which contained fecal matter and shot. The disease was of ten years standing, (Traité des Maladies Chirurgicales, Ouvrage posthume de J. L. Petit, tome ii. p. 186, 199, 201, 205.) Stalpart Vander-Viel relates a case of a man, who swallowed the jaw of a fish, and, seven months afterwards, had it extracted from an abscess near the anus, (Cent. ii. Part. i. Obs. 21.) Sherman mentions a case, in which a fish bone was swallowed, and discharged twelve months afterwards, from an abscess by the side of the anus, (Philos. Trans. 1723). Harrison describes a case of abscess, which resulted from the retention of an apple core, eight months after it was taken into the stomach. (Memoirs of the Medical Society of London, vol. v. p. 154. 1796.) M. de la Peyronie extracted a beef bone, M. Febvrier removed a pullet bone, and M. Dubois a piece of an earthen ware pot, from stercoraceous abscesses. (Mémoires de l'Académie Royale de Chirurgie, tome iii. Paris, M,DCC,LXXXI. p. 124, 126, 128, 129.)" 238.

*Treatment.*—When an acute phlegmonous abscess, with or without disease of the rectum, is about to form near the anus, the patient should be kept in the recumbent posture, and leeches, poultices, &c., applied. As soon as there is any thing like fluctuation, the abscess should be evacuated by an opening.

When the abscess is subacute, the treatment may be of a less antiphlogistic character. Dr. Bushe recommends the early discharge of the matter, but advises this being done by two or three small incisions, rather than by a single free one. We will not pronounce on the superiority of this plan in so confident a tone as Dr. Bushe. The reasons that he gives are not conclusive. In this way, he says, the matter can be freely evacuated, at the same time that the danger arising from the introduction of air, and erysipelatous inflammation, is avoided, as well as, inversion of the lips of the wound, and consequent difficulty in healing it.

The acute gangrenous abscess should *not* be treated by active antiphlogistic measures. Light cathartics, light nourishment, an *early* incision, opium perhaps in the early stage, support by diet and by medicine in the later ones, are the remedies which almost naturally occur to the mind of the practical surgeon.

"Should the skin and cellular tissue be destroyed, as described at page 234, the tonic course, which we have now mentioned, ought to be continued, until the health improves, when it will be necessary to perform an operation to remedy this state of disease; for the contraction of the sphincter, by separating the intestine from the walls of the pelvis, will render reparation impossible. The operation, which I have performed in these cases, consists in passing Desault's wooden gorget into the anus, and then carrying the bistoury into the chasm, I divide the sphincters on it, to the verge of the anus.* Should both sides be similarly

---

* "No matter how far the rectum may be denuded, we are not justified in extending the incision beyond the upper edge of the internal sphincter. Those who think it necessary to divide the rectum, when an abscess forms by its side, take especial care to extend the incision to the bottom of the chasm. The impropriety of touching the rectum, under such circumstances, I have before explained, in note to page 240. Therefore, it now only remains for me to show the absurdity of dividing so much of the gut. My own opinion is, that however ingenious M. Faget may have been in surgical expedients, he was not much of an anatomist, as may be deduced from his statement, that the bowel at the bottom of the abscess is surrounded by bunches of circular fibres, (Op. cit. p. 261.) Now, if this were the case, his practice would be more excusable; but, the fact is that, above the internal sphincter, the circular fibres of the rectum are neither particularly strong nor numerous, (see page 12,) and no anatomist ever entertained the most remote idea, that the pouch of the rectum was in a state of contraction, except during defecation. Even Dr. O'Bierne, who contends so strongly for the contracted state of the rectum, admits, that the pouch does not partake of this condition. To be short, as the object in dividing the rectum, is to prevent its contraction, and consequent separation from the walls of the pelvis, it is unnecessary to divide more than the portion, which, under ordinary circumaffected, I treat them in the same manner. I then pass a ligature through the angle of each flap, and plug the intestine with lint. Finally, I fasten the threads, by means of adhesive plaster, to the buttocks. In this way, the gut is prevented from retracting, while it is well pressed out towards the hips. In a few cases, I have omitted the ligatures, but not without having had to regret it. For some time after this operation, the patient should live upon the most meagre diet, so as to render the feces as scanty as possible, and the bowels ought to be quieted by small doses of laudanum. Every three days, however, we must remove the dressings, and wash out the rectum with an enema of gruel and oil." 244.

If there is a foreign body present, it ought, of course, to be extracted, and in every case, the lower extremity of the rectum should be cautiously examined, in order to ascertain whether there is a foreign body or not.

Mr. Syme's recommendation with regard to the treatment of abscesses in the neighbourhood of the rectum are brief, but to the point.

When the formation of matter, he says, in the vicinity of the anus is threatened by the occurrence of pain, hardness, or swelling of the part, it is usual to abstract blood locally by leeches or cupping. Some relief may thus be generally obtained,—but the improvement, is neither complete nor permanent, and the progress of the complaint, though it perhaps becomes more slow, is not less troublesome,—being rendered sluggish and unmanageable. The application of heat and moisture by means of the hip-bath or fomentations has a very soothing effect on the patient's uneasy feelings, and accelerates the termination of his complaint, either by inducing resolution of the inflammatory action, or promoting suppuration. Evacuation of the bowels should be facilitated by the administration of gentle laxatives, such as castor oil, and injections of warm water into the rectum ; and the patient must confine himself to the horizontal posture, as well as the antiphlogistic diet, with strictness in proportion to the acuteness of his symptoms.

So soon as fluctuation can be perceived, it is considered right to evacuate the matter, which otherwise might diffuse itself into the neighbouring loose cellular texture, and lay the foundation of troublesome sinuses. The knife is now almost exclusively employed for this purpose, and a free incision is made by it from the hip towards the anus, through the centre of the undermined integuments. Poultices are then applied for a few days until the inflammatory engorgement subsides, after which the cavity gradually contracts, and the case passes into the condition of a sinus or fistula. It might be thought better to divide the septum between the abscess and gut in the first instance, and some practitioners have advised this to be done. But it appears that recovery after the operation is not so speedy or so certain when it is performed thus early as when it is delayed until the textures affected are allowed to subside into their natural state.

Dr. Bushe's observations on some other points contain nothing worthy of notice.

2. *Fistula in Ano.*

The mode in which abscess of the cellular membrane by the side of the anus terminates in fistula is well described by Mr. Syme.

---

stances, is in a state of contraction; hence, the incision ought not to extend higher than the upper edge of the internal sphincter."

"In the first place," he says, "a collection of matter is formed under the integuments of the hip near the anus, and usually to one side of it. This deposition sometimes occurs quickly, with heat, redness, and pain of the part, at other times slowly and insidiously, without any sign of inflammatory action, so that the first circumstance which attracts attention is a flat and ill-defined swelling that results from the presence of the fluid, together with thickening of the adjacent cellular substance. In whichever of these ways the abscess is formed,—and every variety is met with, from the rapidity of a few hours to the slowness of as many months,—the matter, if left to itself, sooner or later, by inducing absorption of the neighbouring textures, makes a way for itself to the surface. But as it is situated between the skin of the hip and the mucous coat of the rectum, it may effect evacuation through either the one or the other of these coverings. In conformity, however, with the general law as to progressive absorption occasioned by the pressure of matters foreign to the healthy constitution of the body, it most frequently escapes by an aperture through the external integument. This opening is usually very small, often hardly perceptible, and if the cavity be examined after its contents have been discharged, the mucous membrane will be found almost completely denuded, to a small part of its extent, at the distance of an inch or a little more from the anus. As the matter to get into this situation would, if originally deposited externally to the sphincter, have to penetrate between the muscular fibres, its formation probably takes place in the vicinity of the inner coat of the bowel, whence it proceeds outwards, overcoming the obstacles opposed to its progress in this direction, instead of pursuing an inward course, in opposition to the general tendency which leads to the external surface of the body.

If the patient has been previously in pain he feels comparatively well after the matter is evacuated, and may suppose that he is to recover without any farther trouble. But the cavity of the abscess, though it contracts, does not become obliterated; the discharge continues of a thin and watery consistence; and the orifice acquires a still greater degree of straitness, at the same time generally projecting from the surface of the skin in the form of a small pimple-like protuberance, at the summit of which it is situated. This appearance is owing to an effusion of organizable matter round the opening, in consequence of the continued irritation which is caused by the discharge passing through it. From the same cause the sides of the sinus acquire an increase of thickness and density so as to assume the condition which in surgical language is designated fistulous. If the disease be still permitted to pursue its course unchecked, a small aperture is sooner or later generally formed also through the thin denuded part of the mucous membrane of the rectum. It may seem surprising that this second opening should be formed after the matter has procured vent elsewhere; but there can be no doubt as to the fact, and it agrees completely with what is observed to happen in the case of abscesses situated in the neighbourhood of the urethra, which, after their evacuation, whether spontaneous or artificial, often discharge purulent matter alone for a time, and then urine also." 8.

This is an account of the ordinary manner in which an ordinary fistula is formed. There are several forms of it, and they deserve attention. Mr. Syme observes with truth, that the apprehensions of the public on the subject of fistula in ano, or, at all events, on that of the operation required for it, are preposterously exaggerated. They are derived, in fact, from what the operation was, rather than from what it actually is. Mr. Syme quotes from Dionis, some circumstances which may interest our readers.

"Louis XIV. suffered from *fistula in ano*, and, being naturally unwilling to undergo the operation which his medical attendants assured him was necessary, listened to various proposals for curing the disease without having recourse to the knife. Instead of trying these methods on his own person, however, he collected a great number of his subjects who laboured under the same infirmity, and caused the proposed experiments to be tried on them. Some of them he dispatched to the waters of Bareges, others to those of Bourbon, and many more he shut up in rooms provided with everything that could be suggested for the purpose in view. At the end of a year, finding that not a single patient had been cured, his Majesty yielded to necessity, and permitted his surgeon, M. Felix, to perform all the incisions which he judged proper.

We have here a striking illustration of the necessity of the operation; and the importance attributed to its performance as formerly practised, may be estimated from the number of medical men who were present on this occasion, together with the amount of their remuneration. Besides the surgeon and assistant-surgeon, there were two physicians, four apothecaries, and an apprentice, and the sum total of their fees was L. 14,700." 3.

Those were royal days for surgeons. Had M. Roux the honour of cutting the present King of the French, we question whether Louis Philippe's bounty would swell to the sum of £6000.—the fee which the operator on the Grand Monarque, M. Felix, pocketed. The name of this gentleman is emblematic of the palmy days of surgery in which he had the luck of flourishing. If an operator now receives a thousand pounds for giving sight to one of our millionaires, he is thought a marvellously fortunate fellow.

But we were speaking of the varieties of fistula. As Dr. Bushe observes, they have been divided into three classes, *viz.* those which communicate with the gut, and open on the cutaneous surface; those which, though they communicate with the gut, do not open externally; and those which do not communicate with the gut, but open externally. The number of external openings vary; generally, there is but one; but sometimes, two, three, or even more.

"There is seldom more than one internal opening; in some, particularly in phthisical subjects, there are two; and M. Ribes, who investigated this subject with the greatest care, says that he once observed three. Until the researches of this accurate investigator were published, surgeons were of opinion that the fistula frequently opened into the intestine, at a great height, a mistake which led to a severe and hazardous operation. He, however, demonstrated that the internal orifice was generally situated immediately above the spot where the internal membrane of the rectum unites with the skin, sometimes a little higher up, but never more than five or six lines. In eighty subjects, the internal orifice did not exceed this height, and in a certain number, its elevation was not more than three or four lines. I can bear testimony to the truth of M. Ribes' conclusions, for in none of nineteen subjects, was the internal orifice of the fistula situated higher than in those examined by this surgeon, and in the many cases I have operated on, I never found the internal orifice higher up, than the region of the internal sphincter, and hemorrhoidal plexus." 248.

The internal orifice, continues Dr. Bushe is sometimes round and callous, especially in phthisical patients. In other cases, and these by far the most numerous, it is irregular and soft. The external orifice in like manner may be round, and studded with exuberant granulations, which readily bleed, particularly when old; or it may be irregular and without granulations, especially when recent, and the result of gangrenous inflammation; for the skin, in such cases, is generally undermined, partially livid, and deprived of its vessels by the sloughing of the subjacent cellular tissue, so that it does not really possess the power of creating granulations. The parts surrounding these fistulæ are generally very hard, and sometimes they are so disorganized, that we trace with difficulty the course of the sinuses. If we examine a recent fistula, we shall find that there is a considerable cavity between its orifices, because the sac of the abscess, which gave rise to it, has not yet contracted. This cavity, however, gradually diminishes in size, until it becomes a simple tube, lined by a fine smooth tissue, resembling a mucous membrane, save that it is destitute of villosities.

The situation of the internal orifice of the fistula is a point of such importance that Mr. Syme, as well as Dr. Bushe, dwells very strongly upon it. The former gentleman adds something to the observations of M. Ribes and of Dr. Bushe, and we quote his remarks upon the subject.

"The importance of this observation" (that of M. Ribes) "will appear when it is recollected that the operation requires division of the parts intervening between the two openings of the fistula; since, unless the internal one be sought for in the proper place, it may escape detection, and thus not only occasion an unnecessarily high section of the septum, but, from not being included in the incision, lead to a continuance of the disease. When the internal opening is sought for at the summit of the sinus it cannot be found, so that the fistula is apt to be supposed incomplete or blind external; and M. Ribes, avoiding this error, ascertained that an internal aperture existed much more frequently than had formerly been supposed. He went indeed into the opposite extreme, contending that it was present in every case requiring the operation, and accounting for its constancy by attributing the origin of the disease to ulceration of the mucous coat of the gut. But I have already stated that the abscess which gives rise to fistula is very generally discharged outwards in the first instance; and every attentive practitioner must have remarked that an internal orifice is very seldom met with in recently formed fistulas; which facts are quite inconsistent with this theory of M. Ribes.

I have ascertained farther, that, in those cases where an internal aperture does not exist, the mucous membrane at the part in which it would be situated if present is not only denuded, but rendered so thin that the perception of a probe through it is hardly less distinct than if it had entered the rectum; and that, if the incision extends to this point, the cure will be no less certain than if an opening into the gut had existed." 24.

We now return to Dr. Bushe. The direction and situation of fistulæ, he continues, vary. Both hæmorrhoidal tumors and the stercoraceous abscess which result from the lodgment of fæcal matter in the lacunæ, generate small fistulæ, situated either entirely within the sphincter, or in its substance. The other fistulæ open at a greater distance from the verge of the anus, and extend obliquely upwards and inwards, through the external sphincter, and cellular and adipose tissue, until they open into the gut. In this course they, sometimes, run between the sphincters, and then ascend a little, before perforating the mucous membrane; while at other times they pass through the fibres of the internal sphincter.

The symptoms of fistula are not undeserving of attention, for they are not unfrequently mistaken. There are few surgeons who have not seen instances of fistula overlooked by medical men, sometimes undiscovered even by a direct examination. Mr. Syme mentions a case of this description.

"As has been already observed, the orifice of the sinus is usually very small, and, though generally rendered more manifest by being elevated above the surrounding surface, it still not unfrequently escapes the notice of the patient, who supposes that the discharge issues from the anus. Even the surgeon sometimes experiences difficulty in detecting the disease from this source of obscurity; and I once operated upon a gentleman for a complete fistula, after he had been assured by an eminent physician, who carefully examined him, that there was no morbid affection whatever in the neighbourhood of the rectum." 13.

The symptoms enumerated by Mr. Syme as the most constant, are, uneasiness about the anus, with a more or less copious discharge of thin purulent matter, staining the linen, and otherwise annoying the patient. The occasional escape of flatus and mucous fluid, he continues, from the rectum, are generally superadded in the case of a complete fistula. But the passage of feculent matters through the preternatural channel, though often mentioned as a part of the inconvenience experienced, does not usually take place, and indeed is never met with, except when the disposition to the disease is very strong, as in confirmed phthisis, in which case the aperture of the fistula, external as well as internal, instead of being small and circumscribed by effusion of organizable lymph, is large and flabby. Besides the exudation from the fistula, and more or less uneasiness about the part, especially in going to stool, people of much sensibility are farther distressed by a feeling of weakness and imperfection, which renders their existence almost intolerable. There are other persons of a less sensitive constitution, who, giving themselves no concern about the disease beyond its obvious effects, are able for a long while to endure the discomfort which it occasions. Mr. Syme mentions as an instance of this, the case of a gentleman between fifty and sixty, on whom he operated for a complete fistula with two external openings, which had existed for thirty-five years.

The fluid which is discharged varies both in quantity and quality. It often seems on the point of ceasing, when perhaps another abscess forms, or, at all events, no actual cessation takes place.

"When the fistula opens into the gut, more or less flatus and mucus must pass through it, owing to the resistance which the sphincter muscle opposes to their exit by the anus, and thus adhesion or contraction in the surface of the sinus will be effectually prevented. But when the fistula is not complete, the reason why it should not heal like a sinus in any other part of the body is less apparent. The mere laxity of the texture, or any other peculiarity in the nature of the part concerned, is not sufficient to account for this, since suppurating cavities in the neighbourhood of the rectum are known to heal very kindly and readily, as for instance that which results from the operation of lithotomy. When the sinus, as it almost always does, penetrates between the fibres of the sphincter, the obstinacy in question may be ascribed to the frequent motion and separation of the sides of the cavity, which must result from the action of the muscle. But even this obstacle to recovery is not always present, since the fistula sometimes lies quite superficially under the skin and mucous membrane, without passing through the muscular fibres at all. In such cases it seems most probable that the detached and denuded state of the mucous coat of the gut impedes the healing action." 15.

*Exploration of the Fistula.* This is of some consequence, we mean that the mode of performing it is of consequence. Fistulæ are frequently overlooked, not merely on account of the smallness of the external orifice, but from the imperfect examination and inconvenient position of the patient. Dr. Bushe's directions are sufficiently explicit.

To examine a patient, he says, he should be placed leaning over the back of a chair, or in the position for lithotomy; the buttocks being separated by an assistant, the surgeon ought to search for the external opening. If the fistula be large, and complete, he will find it at some distance from the anus; but, when small, it may be concealed in the folds of the fine skin close to, or at this orifice. Then, having oiled, and gently introduced his fore-finger, he should take a probe of large size, if the external orifice be far from the anus, and small, if in the folds of fine skin, and introduce it gently, rather in a transverse direction, varying it's point according to the resistance it receives. In this way, if there be an internal orifice, he will soon discover it. Should the surgeon direct the probe more upwards, he will elevate it above the internal orifice of the fistula, and as the least force will be sufficient, especially in recent cases, to carry the probe onwards, through the walls of the sinus into the cellular tissue, by the side of the gut, he will be impressed with the idea, that he is only pursuing the trajet of the fistula; and when he cannot find the internal orifice, he attributes his failure to the great height of the opening. Dr. Bushe confesses that he has himself committed this mistake, and, probably, there are few who have not committed it too.

" Should we be unable," he continues, " to discover an external opening, we may suspect that there is an internal fistula, if there be difficult defecation, and the feces are streaked with matter. These symptoms, however, may depend upon other diseases of the rectum and anus; but they are sufficient to warrant a very careful investigation. The parts adjacent to the anus ought to be cautiously examined, and should this disease exist, we will generally be able to discover some induration, perhaps fulness, and, by pressure, matter will issue from the anus. When the finger is introduced, a depression marking the site of the orifice, can in most cases be discovered. If the fistula be small, concealed between the folds of the mucous membrane, and communicates with a small purulent sack, situated within the sphincter, it may escape observation, and the patient be tormented with the most agonizing suffering. I once saw a case of this kind in a man, who had consulted various surgeons, and whose sufferings were as great, as those of any patient I have seen tormented with fissure. When he strained forcibly, and I made pressure on the verge of the anus, I perceived that matter issued from a point a little above; therefore, I passed a curved lachrymal probe into the sinus, which I laid open, and thus afforded him relief. I have seen some cases, in which there were several of these small fistulæ." 252.

The disease being detected, the next consideration is—how to cure it.

*Treatment.* A variety of methods have been at different times resorted to for the cure of this disease. Injections, setons, extirpation of the sinus, division of it, nay, in the hands of the renowned Mr. Van Butchell, stitching it up with a needle and thread, are some of the numerous modes of procedure. We have no intention of examining these in detail, or of resuscitating buried operations. It is sufficient to point out the plans which are found to answer the best.

1. When the sinus does not open into the gut, an operation may be frequently dispensed with. M. Ribes, indeed, says that, under such circumstances, it is never necessary. As Dr. Bushe remarks, this opinion is decidedly incorrect.

Compression made with a piece of cork supported by an elastic T bandage,

—the injection of a solution of the sulphate of zinc or copper, or nitrate of silver, of yellow wash, or port wine,—the horizontal position,—and, an easy state of the bowels, are generally sufficient for the cure. We have cured two cases by the introduction, twice a week, of a probe, the end of which has been immersed in melted nitrate of silver. A small film of the caustic adheres to the end of the probe, and, by means of the latter, can be carried to the bottom of the sinus.

When however, says Dr. Bushe, the cellular tissue has been extensively destroyed, we will sometimes be compelled, after the failure of the means mentioned, to divide the external, and, perhaps, a portion of the internal sphincter. This may be accomplished with a sharp-pointed straight bistoury, armed with a small ball of wax, on Desault's gorget, or, we may introduce the finger into the gut, and a probe-pointed bistoury with a projecting cutting edge, into the purulent chasm, and when the extremity of the instrument has arrived at the upper edge of the internal sphincter, provided the chasm be so deep, we should steadily cut on the nail of the finger which has been introduced into the rectum. Then, turning the finger round, and pressing it on the end of the bistoury, we ought to depress both hands, and thus divide the intervening parts. Some surgeons prefer performing this operation with Savigni's double-bladed bistoury. When this instrument is used it should be introduced with the sharp-pointed blade concealed, until it arrives at the spot about to be transfixed; then, while the instrument is held steadily, the sharp-pointed blade ought to be projected through the gut, and immediately withdrawn, so as to enable the surgeon to pass the united blades through the orifice thus made, and to complete the operation, as when he employs the single probe-pointed instrument.

We must say that we think Savigni's a very clumsy and a very unnecessary instrument. We have found the common probe-pointed bistoury open to a serious objection. The size of the button at its end opposes such an obstacle to its being pushed through the wall of the rectum, that the surgeon is compelled to use a prejudicial degree of force. This tends to separate the tunics of the gut from the surrounding parts, and, (so we have found or fancied that we found,) to lay the foundation for an extension of the sinus.

Having occasion not unfrequently to operate for fistula, we had, some years ago, a probe-pointed bistoury constructed, with as narrow a button and blade as are consistent with the requisite strength of the instrument, and with the prevention of a puncture on the operator's finger. We have found this instrument answer well, and we observe with pleasure that Mr. Syme has adopted, and represented in his book a bistoury of a similar description.

We return to Dr. Bushe.

" In some of these cases the integuments are livid and cribriform. They are badly supplied with blood, in consequence of the sloughing of the cellular tissue, and no effort that we can use, will cause the chasm to fill up. The proper course under such circumstances, is to remove the diseased integument, and if then, after we have dressed the parts properly, the chasms do not fill up, we will be compelled to divide the sphincter. I have seen a great many cases of this kind, and have verified, repeatedly, the justness of what I have asserted. In some instances, I have merely divided the diseased integument, throwing the different openings into one, and extending the incision forwards, backwards, outwards, and inwards, to the union of the integument with the surrounding parts, but in all these cases I have afterwards been compelled to remove the angles of the flaps, as they curled up, became indurated, overlapped the wound, and in some instances cicatrized internally."

2. When the fistula is complete, the surgeon should remember the fact insisted on by M. Ribes, that the internal or rectal orifice is never at a greater height than an inch and a quarter. Extensive and deep incisions, fraught, as they confessedly are, with the risk of serious hæmorrhage, must be nearly, if not altogether unnecessary. Mr. Syme quotes the opinions and criticises the practice of Sir A. Cooper, Mr. Copeland, and others—opinions and practice founded on ignorance of the fact insisted on by M. Ribes. Mr. Syme concludes—

"If the case be as I have stated it, the opinions and practice of which these quotations afford a specimen must tend to occasion great unnecessary suffering; and, therefore, believing that I have not in any respect exaggerated the benefits which are derived from the principles at present advocated, I think it right once more to state them.

1. In complete fistula, the internal opening does not lie farther from the anus than an inch and a quarter, and is frequently much nearer to it.

2. In external fistula not communicating with the gut, the mucous membrane is denuded and attenuated at the part where the opening would be if there were one.

3. In performing the operation it is merely necessary to divide the parts lying between the external and internal apertures, or denuded part of the mucous coat corresponding to the latter.

4. In the after-treatment it is not necessary to interpose any dressing between the edges of the wound beyond the first forty-eight hours." 30.

It may admit we think of question, whether these propositions, especially the latter, are not too unconditional. We do not perceive how the abandonment of dressings after forty-eight hours, follows as a logical sequence of the premises, and in point of fact we do conceive that we have witnessed good, not evil, from light and judicious dressings to a much later period.

The mode of operating for complete fistula is briefly, but sufficiently described by Dr. Bushe. The surgeon should introduce his right or left fore-finger into the anus, according to the side affected; then, with a probe-pointed bistoury, he ought to traverse the sinus, and having placed the finger *in ano* on the extremity of the bistoury, he should cut his way out, either by steadily depressing both hands, as before described, or else by projecting the knife through the anus, and pushing it downwards, and to the opposite side. If the operator be inexperienced, he may first pass a director, and on it the bistoury.

Mr. Syme adds a hint or two. When much difficulty, he remarks, has been experienced in finding the internal opening, it is a prudent precaution, especially for a surgeon not much practised in the operation, to push the probe through the sinus, and bring its point out at the anus, before using the knife, since it is thus impossible to miss the orifice by transfixing the thin membrane which surrounds it. If any sinuses extend under the integuments of the hip or perineum, they should now be laid open with the knife, and then small pieces of dry lint are placed between all the cut edges.

3. When the fistula does not open externally, the surgeon, says Dr. Bushe, may follow one of two methods, in the performance of the operation. In the first, the orifice being discovered by the finger in ano, the operator should carry the knife used for fissure along his finger, and fixing its extremity in the orifice of the fistula, he ought to cut outwards, dividing the sphincter, &c. In the second, having hooked a strong probe, and passed it into the fistula, he should press it down until it appears by the side of the anus, and then cut on its extremity, so as to convert the incomplete into a complete fistula; after which he ought to finish the operation with a probe-pointed bistoury, as above-described. If this species of fistula be small, and situated between the sphincter and mucous membrane, or in the sphincter itself, we should use the small sharp-pointed bistoury.

*After Treatment.*—The following are the recommendations of Dr. Bushe upon this score:—

After, he says, these different operations for fistula, so much lint as will prevent the adhesion of the lips of the wound, should be introduced, and ought not to be removed by the surgeon, but allowed to come away with the fæces. The patient ought to be confined to his bed, and his diet should be as meagre as possible. On the third day, a dose of oil should be exhibited, and after its operation, the wound ought to be cleansed, and a saturnine poultice applied. On each succeeding day, until the wound is nearly healed, the surgeon should inject a little gruel and oil into the rectum, so as to procure an easy evacuation. On the fifth day, suppuration will be fully established, and generally the inflammation sufficiently subdued, to permit the introduction of a very small dossil of lint into the bottom of the wound; a practice which, when repeated a few times, insures the healing from the bottom. At one period, surgeons followed the hurtful practice of cramming the wound with lint, but for some years past, they have stepped into the opposite extreme, introducing but a very small quantity of lint, applying but one or two dressings, and insinuating a probe occasionally, between the lips of the wound, so as to prevent adhesion. Should bleeding follow the operation, gentle pressure will generally be sufficient to check it. If this fails, we may try the application of ice; but if necessary, we may remove the dressings, and introduce a blunt gorget or speculum, and thus expose the bleeding vessel, which ought to be seized with a forceps, and tied.

Should the wound be slow to heal, we may exhibit Ward's paste or cubebs, and apply a lotion of the sulphate of zinc, sulphate of copper, or nitrate of silver, or we may prefer the ointments of the oxide of zinc, superacetate of lead, white precipitate or nitrate of mercury.

This concludes what Dr. Bushe has to say upon fistula in ano. Mr. Syme adds some further observations.

1. *Complication of Fistula with Phthisis.* Mr. Syme touches lightly upon this. The surgeon should certainly endeavour to ascertain if pulmonary disease is present or not in any case of fistula on which he may propose to operate. If pulmonary disease, or a strong disposition to it, exists, the fistula, as a general rule, should not be cut. But if it is producing serious inconvenience, and injuring the patient's health, we conceive that the surgeon is justified in operating. It must be remembered, that pulmonary disease occasionally becomes quiescent, and a patient who appeared rapidly hurrying to the grave, may unexpectedly be restored to comparative health. Local diseases, such as fistula, scrofulous affections of the joints,

&c., appear in some instances to act beneficially on diseases of the lungs, and to operate as a sort of derivative remedy. But in others, the minority, they seem to aggravate pulmonary affection by imparing the constitutional powers, and their removal is beneficial to both.

It must be confessed, however, that the operation for fistula and its cure, is more frequently succeeded by pulmonary mischief than improvement. We have operated several times on patients, who, at the period of the operation, displayed no signs of disease of the lungs, but have afterwards been attacked with it, and sunk under it. The majority of surgeons must have witnessed the same thing, and hence the general misgiving with which the operation is regarded. Still, under all circumstances, not absolutely unfavourable, we are bound to give the patient the chance, perhaps a fair one, which the operation offers.

2. *Urethro-rectal Fistula.* We extract Mr. Syme's remarks upon this head. He presents a fair account of this complication of fistula.

"Fistulous openings near the anus, and leading into the rectum, sometimes communicate also with the urethra. The origin of this complicated form of the disease is an abscess situated between the prostate gland and perineum, which, from not being evacuated early by incision, discharges its contents into the urethra and rectum, before overcoming the resistance to an outward course, which is opposed by the fascia of the perineum; and when at length openings do take place in the skin, they are usually situated at the verge of the anus and root of the scrotum. Flatus and thin feculent matter escape by the urethra, urine issues from the rectum, and a copious fetid discharge proceeds from the external orifices. The patient suffers great and unceasing distress, and, unless relieved by efficient treatment, ultimately sinks under the continued irritation and exhaustion.

These formidable consequences of allowing the abscess to open spontaneously render it incumbent on the surgeon to be careful in recognizing the disease at an early period, and giving free vent to the matter by an ample incision through the integuments and fascia of the perineum. The disease is generally induced by exposure to cold. It commences with pain in the region of the prostate gland, aggravated by micturition and going to stool, and is attended with more or less fever. When the matter begins to accumulate, difficulty is experienced in voiding the urine, sometimes to the extent of complete retention, and requiring the catheter to be introduced. The patient may continue in this state without any alteration, except the occasional occurrence of rigors, for eight or ten days or even longer, until the fluid makes a way for its escape. The perineum when examined is found to be fuller than natural. But, as the integuments retain their ordinary colour and consistence, this change may readily escape observation; and fluctuation, owing to the depth of the abscess, can hardly be perceived unless the finger is introduced into the rectum, through the coats of which the fluid is easily felt. I have frequently been asked to draw off the water when obstructed in this way, without any suspicion having been excited as to the cause of difficulty, and have known the practitioner first take alarm from observing the catheter contained pus. Examination by the rectum, together with the history of the case, will leave little room for doubt as to the existence of matter. But if there should still be any uncertainty, it will always be right to make an incision in the perineum, since this can do no harm, and the withholding of it exposes the patient to the danger of all the distressing consequences that have been mentioned, as resulting from spontaneous evacuation of the abscess.

When the disease has advanced to its fistulous state, it is necessary to lay open all the sinuses; and even then the recovery is not always speedy or complete. The operation should be commenced by dividing the septum between the gut and the cavity left by the abscess. For this purpose the knife is introduced into the orifice which lies nearest the verge of the anus, guided upwards until it enters the gut, and then carried outwards through the septum, which in this case is generally more extensive than in an ordinary fistula, from the internal orifice being seated higher, even above the inner sphincter. The sinuses which extend between the anus and scrotum are next to be laid open, and then pieces of dry lint are inserted between the cut edges. The deep incisions which are sometimes required expose the patient to the danger of hemorrhage; and if there should be any appearance of this the bleeding vessels are if possible to be tied, or cold applied to the wound while the hips are elevated, which means seem far more effectual than pressure, owing to the looseness of the textures concerned. After the cure appears to be complete, a very small fistulous communication is apt to remain between the urethra and rectum, allowing a few drops of urine to pass occasionally. If this does not close within a moderate time, or proves annoying to the patient by exciting his alarm, a red-hot iron wire should be introduced into the orifice, exposed by a speculum, as often as may be necessary for inducing contraction and obliteration of the slender canal. In all cases of this kind, especially those which have been long established, it is proper to search the urethra and rectum for stricture; since this additional complication is not unfrequently met with, whether as a cause or a consequence of the fistula it is not always easy to determine." 41.

3. Mr. Syme alludes also to the occasional connexion of fistula in ano with stricture of the rectum. The rectal orifice of the fistula is, he asserts, in the usual position, about an inch from the anus, and consequently below the stricture. The operation is, quoad the fistula, the same as if stricture did not exist.

4. Sinuses near the anus may proceed from caries of the sacrum, or from exfoliations of the ischium, or os pubis. In such cases, the caries or the exfoliation must be cured before the sinus will heal. Mr. Syme mentions two cases which are brief, but not uninteresting.

"About ten years ago I was asked to see a young man who had suffered several operations for what was supposed to be fistula, without obtaining relief, and had at length become exhausted beyond the hope of recovery. A careful examination led to the discovery of an exfoliation lying inclosed in a capsule of cartilaginous firmness, formed by the origins of the flexor muscles of the knee, from the tuberosity of the ischium. After its extraction, the patient quickly recovered, so as to marry and have a family. I lately saw a young woman who had suffered from *fistula in ano* for five years, and wished to have the operation performed. On introducing the probe I felt it grate past a hard surface, and extracted a thin scale of bone, which had probably been detached from the arch of the pubis, as she attributed her complaint to a strain sustained in hastily descending from the top of a coach." 45.

About four years ago we had a case of this description. A young woman had a sinus, opening externally near the anus, and appearing to be a common fistula ani. It had been operated on once without success by a surgeon in the country. On examining her, we found that the probe could, by management, be got to pass towards the ascending ramus of the ischium, on which it appeared to touch exposed bone. We introduced to this point a probe armed with melted nitrate of silver, every third or fourth morning, and on the intermediate days we injected Bates' red wash. The sinus healed perfectly.

This concludes what we have to say on the subject of fistula in ano. We proceed to:—

B. Contraction of the Anus.

This forms the subject of a short chapter, the twenty-fourth, of Dr. Bushe's book. It is not treated of specifically in that of Mr. Syme.

This condition of the anus, observes the former, may be produced; firstly, by the approximation of portions of skin naturally at a distance; secondly, by the deposition of lymph in the submucous cellular tissue, constituting a species of ring around the anus; thirdly, by the effusion of lymph on the surface of the mucous membrane, which frequently assumes the form of filamentous bands; and fourthly, by a process of disorganization, manifested by irregular thickening, cartilaginous induration, and partial ulceration of the fine skin and mucous membrane extending upwards, sometimes for more than an inch.

The first species results from excision of hæmorrhoidal and other tumors, the second and third from inflammation, while the fourth arises from the syphilitic poison.

Sometimes, in consequence of the contracted state of the anus, defecation gives rise to longitudinal laceration, or "fissure" of the mucous membrane. The introduction of the finger occasions great pain, but determines the nature of the disease.

"In the treatment of this disease the bowels should be kept soluble with castor oil, lenitive electuary, or emollient enemata, and the diet ought to be the same as in stricture of the rectum; should there be inflammation, leeches and fomentations will be necessary; when there is a fissure, the sphincter must be divided; if there be hemorrhoidal tumours, they ought to be removed; and if there be much pain and nervous irritation, anodynes will become necessary. In all cases the anus ought to be dilated with bougies, and here I would observe, that great caution is even more necessary than in stricture of the rectum, especially when the disease is cutaneous, else much pain, weight in the loins, abdominal distress, and disturbance of the general health will ensue. When the contraction is the result of disorganization, produced by the syphilitic poison, anti-syphilitic remedies should be employed, though they are, for the most part, inefficacious, as such affections are generally fatal." 259.

Dr. Bushe relates several cases. We shall select two or three.

*Case.* Mr. D. complained to Dr. Bushe, in the latter end of 1829, of difficulty in defecating. He said, that he had been subject to piles for several years; but that, until within ten months, he could always evacuate his bowels easily. Dr. B. found the anus exceedingly narrow, the contraction apparently depending on infiltration of the subjacent cellular tissue. Dr. B. recommended the introduction of the bougie, and emollient lavements, both of which he used as directed, so that, in a few weeks, the parts had regained their natural condition.

*Case.* An officer who had endured great fatigues in campaigning, became the subject in succession, of hepatitis, dysentery, ague, and dyspepsia. By proper medical treatment, and great attention on his own part, he improved much, but never regained his former state of health. In 1824, he contracted an ulcer on his penis, which healed with great difficulty, and was soon followed by secondary symptoms, under which his health rapidly deteriorated.

When Dr. Bushe saw him, in 1826, he was greatly emaciated, with nodes on his bones,—an eruption on the skin,—chronic iritis,—and induration, thickening, and partial ulceration of the marginal integument and mucous membrane of the anus. He had suffered most annoyance from this last affection, having much purulent discharge, constant tenesmus, and excruciating torture, both at and after stool. Leeches, fomentations, saturnine and opiate poultices, the introduction of meshes of lint besmeared with lard and the extract of belladonna, as well as emollient and anodyne lavements, were tried in vain, at the same time that sarsaparilla and the oxymuriate of mercury were administered. He sank, however, in a few months, and, on dissection, about an inch and a quarter of the extremity of the gut were found diseased.

### c. Stricture of the Rectum.

It is in this disorder that quackery rejoices. Occurring out of sight, (if the quack may be trusted, out of reach also), the charlatan lies with comparative impunity, and trusts to darkness to shroud his doings. Though stricture of the rectum is seldom seen after death, it is wonderfully common during life. A patient has constipated bowels, he naturally applies to a rectum doctor, the doctor takes a long bougie, it hitches of course at the lateral turn of the rectum, or higher up than that, the case is one of confirmed stricture, the patient is doomed to be fleeced. Woe to him or her if he or she is rich, for it is rich people who have stricture of the rectum. Once in the hands of the charlatan and deliverance is afar off. The doctor takes care to insist on the necessity of employing some one who understands the introduction of long instruments, and he naturally and properly congratulates the patient on his fortunate application to *him*. The bait too often takes, and the stricture is a confirmed one, so long as it *pays*.

We turn from this disgusting exhibition, to the consideration of disease as it is, and not as cupidity would make it. Stricture of the rectum forms the subject of the twenty-fifth chapter of Dr. Bushe's work, and of the fifth of Mr. Syme's.

#### 1. *Spasmodic Stricture of the Rectum.*

We are not alluding to spasmodic contraction of the external sphincter, a comparatively common and well-known affection, but to spasmodic stricture of the rectum itself. Dr. Bushe long doubted whether such an affection existed, but his doubts were dispelled by the occurrence of two cases, which he relates.

*Case* 1. Mrs. C., an exceedingly nervous lady, for seven months had suffered under weight and pain in the loins, fullness of the lower part of the abdomen, flatulence, want of appetite, and sourness of the stomach. She scarcely ever had a motion without taking medicine, and her evacuations, when of a certain consistence, were small, figured, and voided with difficulty. On examining the rectum with the finger, every thing appeared natural: but, when she stood erect, and strained forcibly, Dr. Bushe was enabled to insinuate his finger into a portion of the bowel, which was considerably contracted. There was neither induration, nor thickening of the coats of the intestine, and by gentle pressure with the finger, the contraction nearly disappeared. Dr. B. ordered her small doses of blue pill, cathartic extract, and ipecacuanha, also, an infusion of calumba with soda; at the same time that he daily introduced the bougie, and allowed it to remain in the gut for an hour. In the course of a week, he diminished the quantity of medicine, and introduced the bougie only every other day. Her dyspepsia disappeared, and in two months, her stools were natural.

*Case* 2. "Mr. H., who was under my care this Summer, was attacked two years ago, after exposure to wet, with symptoms of inflammation of the bladder, which continued, notwithstanding antiphlogistic remedies were employed. He emaciated, became dyspeptic, much constipated, and when the feces were soft, his evacuations were small, and figured. On examination, I found the sphincter ani in a state of spasmodic contraction, as well as a portion of the rectum which the finger barely reached, when he stood erect, and forced down. His bladder and urethra, were in a highly irritable state, and his urine was loaded with lithic acid. By a vegetable diet, tepid hip-baths, soda, pills of the blue mass and cathartic extract, together with the use of the bougie, he recovered in three weeks."

2. *Organic Stricture of the Rectum.*

Dr. Bushe declares that he has not found this complaint a common one:—"The cases I have seen, bore no proportion to the number I ought to have met with, were the statements made in books correct." Dr. Bushe observes also that, independently of the malignant forms of disease, he has very seldom seen a contraction of the rectum, which was not within the reach of the finger. Dr. Bushe adds, in a note containing the opinions of various authors on the site of stricture of the rectum:—

"Before I finish this note, I may mention that, the inexperienced are apt to refer the opposition offered to the passage of the bougie, by the folds of the mucous membrane or the projecting ridge of the sacrum, to stricture of the gut. I am mortified to add, that, I have good reason for supposing there are a few who make a profitable trade of treating dyspeptic patients for stricture of the rectum, asserting that the obstruction is high up, when in truth this intestine is perfectly free from structural disease. Such practitioners, by passing bougies, apparently cure, what in reality never existed, and thus obtain a character for skill in the treatment of this disease, which in truth they do not possess."

This is a subject of such importance, both to the Profession and the Public, that we cannot forbear from fortifying the sentiments of Dr. Bushe by corresponding, and equally strong ones, on the part of Mr. Syme. We may add that most surgeons of good sense and good faith entertain similar opinions.

"The simple stricture is seated very near the lower extremity of the rectum, a little within the sphincter, between two and three inches from the anus. It is here that the gut changes the direction of its course, and after following the curvature of the sacrum, makes a sudden turn outwards to its termination. There is thus formed a sort of angular projection by the posterior surface of the bowel, which may be supposed likely to increase when subjected to continued irritation of any kind, and at length to constitute an inconvenient degree of contraction. It has been maintained that this is not the sole seat of stricture in the rectum, and that the disease frequently occurs farther up the canal, especially at the distance of five or six inches from the anus. Indeed, some have gone so far as to profess their ability not only to recognize, but to treat it successfully when seated beyond the rectum altogether, in the sigmoid flexure of the colon. That contractions of the great intestine may occur in any part of its course, I do not mean to question. But that the thickening and induration of its coats are in such cases usually confined to the narrow limits which constitute a stricture in the ordinary acceptation of this term, or that the strictured part can be accurately ascertained, and efficiently dilated by the use of instruments, I have no hesitation in expressing my unqualified disbelief.

It is very natural for persons suffering from constipation to suppose that obstruction of the bowel is the cause of their complaint; and they are consequently ready to believe in the existence of stricture, when it is intimated to them by their medical attendant, especially if, at the same time, hopes of relief are held out from the employment of mechanical treatment by dilatation. There is too much reason to fear that unprincipled practitioners have taken advantage of this facility in the disposition of their patients to promote their own unworthy views. But I should be sorry to allege, that a want of good faith was requisite either for the discovery or the treatment of strictures high up the rectum. The practitioner is hardly less exposed to deception than the patient; and if he examine the rectum, under an impression that there is a stricture existing in it, he will be very apt to believe that he has found one. In the feeble and unhealthy persons who are usually suspected to labour under the disease, the coats of the rectum are so thin and relaxed as readily to catch the point of the bougie employed for exploring the cavity, and thus impede its progress, which is also apt to be arrested by the promontory of the sacrum. As an instance of this, I may mention the case of an elderly lady whom I saw with Dr. Begbie. She had been supposed to suffer from stricture of the rectum, between five and six inches up the gut, and had been subjected to treatment for it during several years before coming under Dr. Begbie's care, by two gentlemen of the highest respectability in this city. Finding that the coats of the rectum, though greatly dilated, were quite smooth, and apparently sound in their texture, so far as my finger could reach, and conceiving that the symptoms of the case denoted a want of tone or proper action, rather than mechanical obstruction of the bowels, I expressed a decided opinion, that there was no stricture in existence. Not many months afterwards the patient died; and when the body was opened not the slightest trace of contraction could be discovered in the rectum, or any other part of the intestinal canal. One of the gentlemen who had been formerly in attendance was present at this examination; and wishing to know what had occasioned the deception,—which he said had led to more than *three hundred hours* being spent by himself and colleague in endeavours to dilate the stricture with bougies,—he introduced one as he had been wont to do, and found that, upon arriving at the depth it used to reach, its point rested on the promontory of the sacrum. Other cases might be mentioned to illustrate the uncertainty of information as to the capacity of the higher part of the rectum, obtained by exploring the gut, and to show how far the best-intentioned practitioners may be misled by the sources of fallacy I have endeavoured to explain." 113.

The nature of stricture of the rectum is the next point for consideration. Mr. Syme remarks that the rectum, like the œsophagus, which it resembles in many other points of structure, size, and morbid derangement, is liable to stricture of two different kinds. In one of these there is merely contraction of the coats, with thickening and induration of their texture.

But in the other there exists a morbid growth, attended with the symptoms, and prone to the changes, which characterize malignant degenerations of structure. Want of attention to this very obvious and necessary distinction has often led to great misapprehension in regard to the nature of the disease, and serious errors of practice in its treatment. By some it has been looked upon as always admitting of remedy at an early stage, and by others it has been considered always incurable; while the good effect of introducing bougies in cases of the simple or non-malignant kind has encouraged those who supposed the stricture to be constantly of a carcinomatous nature, to expect benefit from the employment of pressure in the treatment of cancer occurring in other parts of the body. These observations are very just.

3. *Simple Stricture of the Rectum.*

Mr. Syme says little on the nature of the stricture. Dr. Bushe relates the particulars of four dissections of it. In one, the lesion seemed to be confined to the muscular tunic and cellular tissue; in another, to the cellular tissue alone; and in two, to the mucous coat and cellular tissue. The alteration of structure, seemed to depend upon the deposition of lymph, which gave to the parts more or less hardness. The extent of the stricture varied from one quarter, to one inch, occupying the entire circumference of the gut, but in two cases, while in one it scarcely passed half around it. In each, the canal was contracted, and especially in one of those, in which the mucous membrane and cellular tissue were involved. So great, indeed, was the obstruction in this case, that it would not permit the extremity of the little finger to pass. The exterior of the intestine in each, appeared to be indented, while the interior was rendered irregular, by the folding or puckering of the mucous membrane; and in the two cases, in which this tunic was diseased, the inequality of surface was still further increased by vascular excrescences, which, in one, projected considerably into the bowel.

In a note, Dr. Bushe informs us that Morgagni gives the case of a woman who died in the Hospital at Bologna, in consequence of disease of the rectum, which, on dissection, was found to consist in a growth of protuberances, from the inner surface of the intestine, commencing six or seven digits from the anus, and extending to within one of this orifice. The coats of the rectum were hard and thick, and the protuberances, which were about the size of large beans, were smooth on the surface, solid and compact in their texture, and in colour and form resembled conglobate glands.

Dr. Bushe also quotes many passages from various authors on the subject of these "tubercles" within the rectum, but, as it appears to us, without arriving at any definite conclusion with respect to them. We therefore waive particular notice of them.

*Age of the Patients.* Dr. Bushe has seen the disease in a lad nine years of age, but the rest of his patients were under sixty, with the exception of one gentleman, who died of it in his seventy-second year. Mr. White observes that the disease is chiefly met with at the meridian of life. Mr. Copeland coincides with Mr. White. Indeed, this is a point on which most surgeons are agreed.

*Sex.* While some authors contend that the female sex is most prone to the disorder, others doubt or deny this. The point is by no means settled. Out of fifteen cases which have fallen under Dr. Bushe's observation, eight were those of women. Mr. Syme simply states that the disease is met with more frequently in females than males, and generally occurs about the middle period of life.

*Predisposition.* Some authors are of opinion that there exists such a predisposition. The point is not of trivial consequence, and deserves a little attention. Mr. White first advocated the opinion, that the narrowness of the colon at the termination of its sigmoid flexure predisposes to stricture. The idea has been eagerly received.

Mr. White says :—" Although it would be absurd so suppose that every case of habitual costiveness proceeded from mechanical obstruction in the passage, yet, from various conversations I have had with different sensible persons, (some medical,) who laboured under stricture of the rectum, I am much inclined to think that the predisposing cause is the gut being somewhat narrower about the termination of the sigmoid flexure of the colon, than it ought to be for the purpose of allowing a free and easy passage to the fæces. There is another circumstance, also, which is deserving of notice; as it has very much tended to confirm the above opinion, respecting the predisposing cause of strictures; and that is, several members of the same family having been afflicted with the disease, which has happened, to my knowledge, in different instances. Such an occurrence cannot, I think, be more satisfactorily accounted for, than by supposing some original malformation in the passage. I think it is not improbable, that sometimes the passage of the fæces may be interrupted, in consequence of an unusual projection of the last lumbar vertebra, or the superior part of the sacrum." 270.

Mr. Salmon has been fortunate in his observations, for he declares that he has "*repeatedly* noticed *several* members of the same family afflicted with stricture." Considering the rarity of the disease, it must be admitted that Mr. Salmon has been remarkably lucky.

The observations of Dr. Bushe upon the point are decided.

" That the normal condition of the canal of the large intestine, at the junction of the sigmoid flexure of the colon with the rectum, disposes to stricture, I deny; because if this were the case, the disease would be much more common than it really is, and though I have spent more than one half of my professional life in anatomical pursuits, I declare that I have never seen a single instance of preternatural contraction of this portion of the bowel, without organic lesion. Nor can I assent to the common occurrence of this disease in several members of the same family, until I either verify it myself, or be assured of its correctness by a disinterested surgeon, adroit in his manipulations, and conversant with pathological anatomy. Finally, when Mr. White alluded to the obstruction caused by the sacrum, he forgot that the intestine had a mesentery at this spot, and consequently fell so far forwards, as to destroy any influence which the sacrum might otherwise have. 271.

*Causes of the Stricture.* Spasmodic contraction of the muscular fibres, inflammation, a peculiar condition of the sphincter ani, the venereal disease, and other causes equally vague, equally unproved, have been accused of producing stricture. Mr. Syme represents, we think, the state of the case, when he remarks that, from the slow and insidious formation of stricture in the rectum, it is not easy to ascertain the circumstances which give rise to it. The analogy of what happens in other mucous canals would lead to the supposition that continued irritation of the gut is probably the immediate exciting cause. But the precise way in which this state is occasioned, or why, when its other effects are so common, it should so rarely produce the effect in question, are points that have not yet been satisfactorily made out.

*Symptoms and Progress.* Both Dr. Bushe and Mr. Syme concur in remarking that the disease not only comes on slowly and insidiously, but, even when established, produces symptoms rather calculated to mislead than to attract suspicion to their real nature. Dr. Bushe's account of those symptoms is more copious than Mr. Syme's, and we shall therefore follow the former.

There is a sense of weight and obstruction in the lower bowels,—uneasiness, distention, and occasional spasmodic pain in the abdomen—eructations —precordial oppression—pain in the site of the stricture, loins, and sacral region, occasionally extending down the extremities,—vesical irritation,—bearing-down in females,—itching and heat about the anus,—headache,—nervous irritability,—and dejection of spirits. The left colon is loaded with gas and fæces, as may be ascertained by an examination of the corresponding iliac fossa. Mr. White pointed out pain towards the occiput as a very common symptom. Dr. Bushe, however, has never seen a case in which the pain was confined to this region. The urine is generally scanty, high-coloured, and fætid, though Dr. Bushe has seen it abundant and limpid.

The bile, continues Dr. Bushe, is also generally vitiated and scanty. When the disease has continued for some time, the hæmorrhoidal vessels become engorged, and very commonly tumors form, which, for the most part, are produced by extravasated blood, and hence it is that, in old cases, the skin about the anus becomes thickened and elongated. In consequence of irritation arising from the stricture, an increased quantity of blood is determined to this region, and its return is so much impeded by the condensation of the walls of the bowel, and the accumulation of indurated fæces, that abscesses form in the cellular tissue, near the anus, and degenerate into fistulæ. The calls to stool are sudden, and amount to six or twelve in twenty-four hours; generally, two, three, or more, take place within a short time, and are accompanied with much straining, which, in some instances, especially when the stricture is situated high up, gives rise to protrusion of the mucous membrane. Much gas and a small quantity of mucus, occasionally mixed with blood, is all that is commonly discharged; but every two or three days fæcal matter, in small pellets if hard, and in long, round, angular or flattened portions of small diameter if soft, is expelled. After each attempt, though the pain is very moderate, a sensation continues as if the bowels had not been emptied, and this is the reason why, in these cases, several evacuations, such as they are, follow one another at short intervals. When, however, a sufficient quantity of mucus or feculent matter has been discharged to give temporary relief, and from habit the amount is very trifling, the patient, who has been fatigued, desists from further attempts, until a sense of fulness indicates the necessity of making another effort. Occasionally, when the fæces accumulate above the stricture, which they frequently do in immense quantity, they are rendered fluid by an abundant secretion from the mucous membrane; in consequence of which, the patient is enabled to discharge nearly or perhaps all the accumulated matter: thus, by an effort of nature, fatal consequences are warded off.

Dr. Bushe alludes, in a note, to a fact mentioned and satisfactorily explained by Mr. White. This gentleman's words are:—"With regard to the lessened diameter of the fæces just noticed, which must necessarily be the case, whenever a permanently contracted state of the gut takes place; yet it has happened, in some instances, where that change had been observed, that in a more advanced period of the disease, fæces of a natural size had occasionally passed. If the stricture should happen to be so low in the rectum as not to allow room for the accumulation of fæces, it must appear that they will be found uniformly small in diameter, while they continue to be discharged in a figured state. And also, when the stricture is high up in the rectum, so long as the gut below retains its natural expulsive powers, an accumulation will be prevented, and the diminished size of the fæces will continue. But, as the disorder increases, the inferior portion of the intestine loses its power; and when the contraction becomes considerable, a small quantity of fæces only pass at a time through the stricture, and not being sufficient to stimulate the lower part of the rectum, an accumulation goes on from time to time, until at length it becomes difficult to remove; and on these occasions, fæces of a natural size have been sometimes discharged." In illustration of this statement, he mentions the case of a clergyman, who died with a stricture in the upper part of the rectum, yet a few days before death, passed a mass of fæces "as large as the natural diameter of the gut."

Mr. Syme dwells strongly on the liability of both patient and surgeon to mistake the disorder, from the circumstance that the effects of a confirmed stricture are in general the frequent, often almost incessant discharge of thin feculent matters, owing to the copious secretion of mucus which results from the irritation of the disease; and that the thin slimy stools, occasionally tinged with blood, attracting more notice than the small indurated masses of fæces passed along with them, make the case assume the appearance of diarrhœa. The mistake thus committed not only prevents the proper means of remedy from being employed, but leads to the administration of astringents and anodynes, which must prove hurtful, by checking the process instituted by the system for its own relief. This consists in the copious secretion of fluids into the cavity of the great intestine, which lessens the solidity of the feculent matters, and facilitates their passage through the narrow channel that remains for their escape.

To return to Dr. Bushe. He remarks that:—

"When the stricture is fully within the reach of the finger, the canal feels narrow, indurated, and unyielding, for a greater or less extent, and in some instances we are able to pass the finger through the obstructed portion. Occasionally, it is rather higher up than we can reach; but in such cases, if the patient bears down forcibly, the diseased portion of the intestine will so far descend, as to admit of the requisite examination. When, however, the stricture is situated still higher up, we should sound the gut with the instrument recommended by Sir Charles Bell, which consists of an ivory ball mounted on a stalk of whalebone. This instrument is much preferable to the bougie, because when the ball is once introduced, the anus is no longer on the stretch, and if there be a second stricture, we can ascertain it with precision: perhaps, I might also add, that, with it the extent of the stricture can be more correctly determined. In consequence of the great tenderness of the stricture in some cases, this examination is attended with considerable pain." 376.

As Dr. Bushe observes, it often happens that years pass by without the patient's general health being impaired. Ultimately, however, he becomes pale, emaciated, and hectic. Purulent matter, so acrid as to excoriate the anus, is now discharged in great abundance, and frequently it comes away when he coughs, or assumes the erect position. These symptoms increase until life is exhausted. Some patients, however, die from the accumulation of fæces, before the disease has arrived at the stage now described—they become melancholy, pallid, excessively flatulent, and breathe with difficulty, in consequence of the resistance to the descent of the diaphragm caused by the distention of the abdomen,—the pulse lose their strength and regularity,—hiccough sets in, and they sink with symptoms of ileus; but before well-marked enteritic inflammation becomes manifest they are generally more or less troubled with cold feet, cramps in the legs, and a determination of blood to the head, all of which arise from the pressure made upon the aorta, or its primitive branches. On examination after death, we find that the intestines are not only amazingly distended, but inflamed, and even partially sphacelated.

We have seen patients die in both these manners. On the whole we should say, from what we have seen, that a not unusual thing is to suffer from occasional attacks of ileus, and, finally, to be carried off by one of them. Mr. Syme mentions an interesting case.

*Case.* "Some years ago I attended a gentleman for *fistula in ano* together with stricture of the rectum. Not long afterwards he told me that his wife complained of symptoms similar to those he had suffered from the latter ailment. I proposed an examination of the rectum, which was declined, and I heard no more of the patient, until raised one night with an urgent request to visit her immediately. She was labouring under the symptoms of peritonitis in its advanced stage, and died before the end of many hours. The rectum was contracted almost to obliteration at the usual part." 117.

Mr. Syme observes that abscesses frequently form in the vicinity of the stricture, so as to lay the foundation of *fistula in ano*. In this case the sinus does not, as has been alleged, open into the gut above the contracted part, but holds its usual position near the anus, and should be regarded rather as an accidental consequence of the neighbouring irritation, than as a direct effect of the stricture.

Dr. Bushe proceeds to state, that, in some very few cases, the stricture is partially destroyed by ulceration; but it is much more common for the rectum immediately above it to be thus affected. In such cases, it generally happens that this intestine becomes incorporated with the bladder in the male, and with the vagina in the female, so that an extension of the ulceration may give rise to a recto-vesical or recto-vaginal fistula, through which the fæces will be partially evacuated. A much more common consequence, however, of the ulcerative process, especially when the stricture is low down, is extravasation of fæcal matter into the cellular tissue, and the formation of stercoraceous abscesses, which degenerate into fistulæ. The number of these fistulæ varies, sometimes there is but one or two, at others from six to twelve, or even more, particularly in women, in consequence of the greater abundance of cellular tissue in the perineum. In some instances, especially when the disease is situated high up, the rectum adheres to another intestine, and by a continuation of the ulcerative process, a communication is established between them; but it much more frequently happens that no such adhesion exists, and consequently that the fæcal matter is effused into the peritoneal cavity, an occurrence which is followed by a rapid and most horridly painful death.

*Diseases simulating Stricture.* These are thus set forth by Dr. Bushe.

"Retroversion of the uterus, enlargement of this organ or of the prostate gland, as well as tumours developed in the vicinity, may simulate stricture, by partially approximating the sides of the rectum, thus rendering defecation difficult, causing figured stools, tenesmus, mucous discharge, fullness, and a sense of weight in the sacral and perineal regions.

Malignant affections, hereafter to be described, have also many symptoms in common with this disease; but the sallow and leaden countenance, the lancinating pains, and the rapidity of the ulcerative process in the former, will enable us to arrive at a proper conclusion.

Ulceration of the rectum, when attended with spasm of the sphincter, fissure of the anus, or spasm of the sphincter itself, may, by the inexperienced, be confounded with stricture; however, the excessive pain which attends and follows the evacuations in spasmodic affections of the anus, will sufficiently mark the difference between them, and that of which we are now treating; yet, it ought to be borne in mind, that spasm of the sphincter may co-exist with stricture.

Finally, painful chronic affections of the vagina may simulate stricture of the rectum, in consequence of the contiguity and nervous association of these organs. The diagnosis, however, is easily determined by an examination.

On the other hand, stricture of the rectum may be mistaken for a sarcomatous tumour, growing into the intestine; for when the feces collect above the stricture, they may so depress the walls of the bowel, together with the stricture itself, as to simulate a fleshy tumour with an extensive base. By searching cautiously, however, we shall be able to discover an opening in it, into which we can insert the tip of the finger, and thus discover the impacted and indurated feces." 279.

*Treatment.* The horizontal posture—light nutritious diet—injections, if they can be borne without inconvenience—mild aperients, if they cannot—anodyne suppositories, enemata, and hip-baths for the relief of pain and irritation—leeches for inflammatory symptoms—such are the obvious means suggested by reason and approved by experience as palliative remedies of more or less utility.

But the dilatation of the stricture must be the work of mechanical operations—of the use of the knife or the bougie, or of both.

Mr. Syme observes that division of the contracted part with a cutting instrument, notwithstanding the obvious risk of hæmorrhage and inflammation incurred by doing so, has been occasionally practised; and with such speedy as well as complete relief, that some practical writers regard this method as the one which ought to be preferred. But as experience has ascertained that, in certain conditions of a constitutional and local kind, wounds of the rectum, even though of very small extent, are followed by serious or fatal consequences; and as the bougie, though not so speedy in its operation as the knife, is equally effectual, and not exposed to the same objection, prudence seems to require that the practice of incision should be either entirely abandoned, or only used in particular cases with extreme caution. The best instrument for the purpose is the blunt-pointed curved bistoury; and the stricture should be either divided backwards, in the direction of the sacrum, or notched at different parts of its circumference by cuts of smaller extent.

Dr. Bushe remarks that, when the stricture is near the anus, narrow and firm, the surgeon may hook it down with his finger, and then partially divide it, in two, three, or more points, with a hernial knife; after which he should insert a short bougie, so as to prevent adhesion of the incisions. This instrument ought to be introduced completely within the anus, and then secured in its place, by connecting a T bandage with the loop of tape attached to its thick extremity. A full dose of morphine should then be exhibited, and the patient put to bed. If there be much irritation and pain after the operation, the bougie ought to be removed without delay, and fomentations or hip-baths, anodyne enemata, and perhaps leeches employed, according to the urgency of the symptoms. Provided, however, that there has been no necessity for any interference, and that a dose of morphine has sufficed to keep the patient quiet, we ought, at the expiration of thirty-six hours, to remove the bougie, free the bowels with an enema, and again replace the instrument; after which, we should proceed with the dilatation in the ordinary manner.

Dr. Bushe adds in a note:—

"I think that many partial incisions are preferable to a single deep one; because they are not so likely to be followed by hemorrhage, inflammation, or adhesion, and the bougie, when introduced, still continues to act on the edge of the stricture; whereas, when there is but one deep incision, the introduction of the instrument causes the edge of the stricture to turn upwards, and the pressure is then directed against its inferior surface. If the stricture be divided high up, and hemorrhage ensues, we must depend upon astringent injections for checking it; whereas, if it be low down, we can dilate the anus with a speculum, so as to discover the seat of the bleeding, and thus be enabled to apply pressure, the cautery, or ligature.

These directions are all important, for if the bougie be allowed to protrude through the anus, it will create so much tenesmus, as to render its removal necessary; and if after it has been passed above the anus, it be not secured, it may be drawn up into the gut, perhaps beyond the stricture." 281.

We think there can be little question of the superiority of several small incisions over a single deep one. Nor can the utility of the former, in many instances, be questioned. But it is only in facilitating the passage and operation of the bougie that division is of service.

Mr. Syme and Dr. Bushe are in a great degree at variance with respect to the action and mode of employing the bougie. They differ on a very important point.

Mr. Syme contends that:—

"The use of bougies in removing strictures is a remarkable example of good practice, originating from false principles. It was at first adopted with the view of destroying the obstruction through the effect of medicinal substances, which were in this way applied to the contracted part of the canal. And when experience had proved that bougies of the simplest composition, as those constructed of metallic substances, were not less effectual than those of the medicated kind, the process of improvement was next ascribed to the mere dilatation acting mechanically as on a tube of dead matter. Hence it was thought impossible to introduce the instruments too frequently or for too great a length of time. At least once a day was thought essential, and they were permitted to remain for hours at a time. But the contracted canal is not composed of dead substance, and the stricture depends upon a peculiar morbid action of the living texture. The beneficial effect of the bougie, therefore, must consist in the excitement of another action opposed to the one formerly in operation, and capable of restoring the gut to its natural state.

It is the effusion of organizable matter into the cellular texture of the part that causes the stricture, and it is the absorption of this deposit which removes the disease. The bougie by effecting pressure excites the action of absorption. And if the pressure be too great, too long continued, or too frequently repeated, there will be a great risk of causing more than sufficient irritation for the purpose; and of inducing again the very condition it is desired to counteract, the consequences of which must be a confirmation and increase of the disease. The perfection of treatment by means of the bougie may thus be considered to consist in using it merely to the extent requisite for producing its beneficial effects; and this is now fully ascertained to be much less than might at first view have appeared possible. Instead of requiring to be introduced daily, and to remain in the passage for hours, it appears that the bougie causes a sufficient degree of excitement if used every third or fourth day, and withdrawn immediately after being passed through the stricture. Under this system the improvement not only advances at least as quickly as when the operation is performed more frequently, but is likewise much more sure in its progress, and much less apt to be interrupted by undue irritation of the part concerned. These principles now regulate the treatment of stricture in all the mucous canals which are subject to it, namely, the urethra, œsophagus, and rectum." 123.

Dr. Bushe states that:—

"The introduction of the bougie in some persons, is attended with more pain and irritation than in others, as manifested by general uneasiness, aching in the loins, shivering, sickness of stomach, and pain in the abdomen; consequently, the frequency of the introduction, and the length of time it may remain introduced, ought to vary in different cases, else peritoneal inflammation, and even death may occasionally occur. In some patients, we shall not only be enabled to repeat the operation daily, but to allow the bougie to remain in the bowel for an hour or more. In others again, once or twice a week will be as often as we dare employ it, and even on such occasions but for a very short time. For the same reasons, we can in some cases increase the size of the instrument much more rapidly than in others." 282.

We cannot altogether agree with Mr. Syme. It is true that the stricture, whether in the urethra or the rectum, is not dilated by mere mechanical pressure, and it is true, also, that such pressure, if pushed too far, is apt to induce an amount of irritation, which increases rather than diminishes the stricture. But we do not think it true that very trifling pressure is as effectual as a fair quantum of it, in promoting the dilatation of the stricture—we do not think it true that the mere passage of an instrument every third or fourth day will produce "at least as quick improvement" as the more frequent passing and prolonged retention of the instrument. Mr. Syme appeals to the urethra. So do we. If we wish a stricture of the urethra to be rapidly dilated we keep a gum catheter constantly in the canal, increasing the dimensions of the instrument as rapidly as we can with prudence. Most surgeons are aware of this, and it certainly makes against Mr. Syme. So far as we should say that in the case both of the urethra and the rectum, it is better, provided we do not irritate the stricture, to pass the instrument frequently, and retain it each time for an hour or two. The limit to the frequency of introduction and duration of retention of the instrument, is the production of irritation. Short of that we do good—if we occasion that, we do mischief.

Dr. Bushe's directions for the use of the bougie are thus given:—

"A bougie of sufficient length to extend beyond the stricture, and so large, as to pass through it without force, should be chosen. The gum elastic and metallic instruments, answer when the stricture is within the reach of the finger, and notwithstanding the abuse which the latter have received, I must say that, I prefer them when the stricture is coupled with hæmorrhoidal tumours; but neither will suit when the obstruction is high up, as they cannot be curved at the moment, so as to insure their safe passage, and even if they could, their firmness would render them very dangerous. In such cases, wax bougies should be employed :* immediately before use, they ought to be immersed in hot water until they become pliant, then well oiled, and curved so as to correspond to the hollow of the sacrum, and lateral inflection of the sigmoid colon.

Previous to the introduction of the bougie, the bladder should be emptied, and the rectum washed out with warm water. The patient may either lie on his left side, lean over the back of a chair, or kneel on his bed, while an assistant separates his buttocks, and the surgeon takes the bougie in his right hand, and introduces it upwards and a little backwards, with the convexity towards the sacrum. If the stricture be more than five or six inches from the anus, he must turn the point of the instrument a little forwards, and to the left side, as in this way he will avoid the sacrum, and best enter the sigmoid flexure of the colon, should he desire it :—yet, to effect this last, it may occasionally be necessary to give the bougie an opposite direction, as the intestine frequently bends to the right side. In passing the bougie, too much care cannot be taken, all force should be avoided, and when it is suddenly checked in its course, it ought to be withdrawn for a short distance, its direction varied, and then passed upwards again." 284.

Mr. Syme does not contemplate the mechanical treatment of the stricture at any distance from the anus. His directions, therefore, have exclusive reference to stricture contiguous to that aperture. Mr. Syme prefers bougies of iron or elastic gum—the former the more durable—the latter the more pleasant. The instructions of Mr. Syme are simple. When the operation, he says, is to be performed, the patient should be placed upon his

---

* "White recommends the bougies to be made in the following manner :—

℞. Ceræ flavæ ℔. 1¼.

Adip. suillæ ℔. iv. m. ft. cerat.

N.B.—In Winter one part of wax will be sufficient to four of lard.

A long piece of lint, folded and tied at one end, is to be dipped in this ointment, and drawn through a wooden mould; when cold, it must be passed through another mould of less diameter; then to be re-dipped, and passed a third time. (Op. cit. p. 173.)

Salmon says, "The bougie is composed of fine linen cloth, heavily coated with wax, and a certain portion of diachylon plaister coloured with a small portion of lamp black." (Op. cit. p. 55.)

I have tried bougies prepared according to both methods, but much prefer those recommended by Mr. Salmon."

side, and then the surgeon, having in the first place satisfied himself as to the precise position of the stricture, by feeling it with his finger, passes a bougie lubricated with oil or lard up to the obstruction, and presses against it steadily but gently. If the resistance cannot be overcome without using force or causing pain, he withdraws the bougie, and tries a smaller one in the same way, thus proceeding until he gets one to pass through the contraction, immediately after which he withdraws it, and concludes the process for that time. If necessary some soothing means, such as an opiate injection, or the hip-bath, may be employed to allay any undue irritation that has been excited even by this cautious proceeding. At the end of three or four days, or a longer interval, if the patient continues to suffer from the former operation, the bougie which was introduced upon that occasion is again passed, and followed up by another of larger size; and thus the treatment is carried on until the disease ceases to occasion any inconvenience, and a full-sized bougie can be introduced with ease.

Dr. Bushe subjoins an account of two modifications of the bougie, which he has adopted.

"For a long time I was greatly dissatisfied with the bougies in common use; because by keeping the anus distended, they caused great pain. To obviate this difficulty, for some years past, I have used bougies three inches long, made of ebony, and mounted on a stalk of whale-bone. An instrument of this kind is not only easily retained, but is introduced with great facility, by inserting the left fore-finger into the anus, pressing the whale-bone stalk a little forwards and to the left side, while with the right hand, it is urged steadily upwards." 284.

The preceding description can be readily comprehended without the aid of plates. The succeeding will not, we fear, be so intelligible. Still it may be understood by any instrument-maker, and a surgeon who is desirous of giving the instrument a trial, may, without much difficulty, get one manufactured. In our own opinion, the apparatus is unnecessary, and might, if incautiously employed, be dangerous. With this brief expression of disapproval, we subjoin Dr. Bushe's account of the instrument.

"Having observed that the pressure, as well as the presence of a bougie in the stricture, was absolutely necessary for the absorption of the effused lymph, I found that in some cases, though I rapidly increased the size of the instrument, but little irritation followed the operation; therefore it occurred to me, that a bougie might be invented, which would dilate the stricture more rapidly, without the risk of lacerating the intestine. With this view, I designed the following instrument, which, however, is not suited to stricture high up in the intestine. It consists of five parts. The first is a silver tube, five inches long, and three-fourths of an inch in circumference, which at the middle splits into four equal parts. The second is a piece of ivory, three inches long, round, varying in circumference, from one and a half to three inches, conical at the extremities, divided longitudinally into four equal parts, each of which is connected with a division of the silver tube, and grooved on its angle: in two points an inch apart, the groove, for an extent varying according to the size of the instrument, from one half to three-fourths of an inch, is deepened in a semilunar form, to within a line of the thickness of the instrument. The third part is a handle, three-fourths of an inch long, half an inch broad, and a quarter of an inch thick, with a worm in it for the reception of the screw. The fourth is a piece of silver wire about a quarter of an inch in circumference, and eight inches long, which runs through the tube, and terminates at one end in a screw, about an inch long, and at the other end in eight semilunar plates, varying from one to three quarters of an inch in breadth, and within a line, as deep as each portion of the ivory is thick. These plates correspond to the pieces of ivory, and fit into the deep portions of the grooves spoken of. The screw of course passes into the worm in the handle. The fifth and last part of the instrument, is a small ring which fits on the silver tube, and serves to keep the split portions *in situ.*

Now, by pulling back the ring, and then screwing the handle, the semilunar plates are retracted from their beds, and the four ivory pieces separated." 286.

Dr. Bushe adds two observations, each of which deserves attention, and both should be balanced against the representations of some interested individuals.

1. He assures us that he has examined several persons in whom the rectum was perfectly sound, yet who had been considered to labour under stricture. Is there any surgeon in moderately good practice who cannot tell a similar tale?

2. He terminates the chapter with this admission:—that though he has ameliorated the condition of many a poor sufferer, he has never cured a single case—that he has known of no patient who was able to leave off the use of the bougie for any time, without an increase, or return of his com- complaint—and, finally, that those who speak of the frequency of their cures must either possess means of which we are ignorant, or have been deceived.

To us it has appeared that those who boast of their cures of stricture, have won their glory easily and cheaply, by removing what never had existence. In short, stricture of the rectum is the golden egg of regular and irregular quacks. We hope that the space we have allotted to the subject, and the manner in which we have dwelt on the real nature and exaggerated representations of the malady, will tend to disabuse some credulous, and to intimidate some designing persons.

*Complication of Fistula with Stricture.* Dr. Bushe observes that, when fistulæ communicate with the bladder or vagina, we cannot interfere with them until the stricture is pretty well dilated; then, we may derive advantage in the first form of disease, from allowing a gum elastic catheter to remain introduced, and in the second, from the actual cautery. Should a fistula open by the side of the anus, and the stricture be low down, we may advantageously lay open both at the same time; but if this be impracticable, we should dilate the stricture with bougies in the first instance, and then operate on the fistula, if it does not heal.

B. *Malignant Stricture, or Carcinomatous Degeneration of the Rectum.*

It is neither consistent with our best principles of nomenclature, nor is it indeed consistent with fact, to term the following alterations "malignant stricture" of the rectum. If stricture occurs, it is an accidental consequence rather than an essential feature of the malady, which is substantially a morbid growth or morbid change of structure, and, as a morbid growth, runs on to the destruction of the patient.

Dr. Bushe remarks that the malignant alterations of the rectum may be cartilaginous, lardaceous, or encephaloid. We cannot, however, consider

Dr. Bushe's description of these changes satisfactory. He says little with respect to them, and that little is vague.

The cartilaginous disease, he observes, may either commence in the form of hard tubercles on the mucous membrane, or in the muscular coat of the intestine, which is by far the most common,—the fibres become pale and firm, while the connecting cellular tissue undergoes a similar process of condensation, without any alteration of colour. As the deposition goes on, the cellular tissue frequently becomes lardaceous; but, however this may be, the walls of the bowel increase in thickness, and the cellular and muscular tunics are sooner or later confounded and softened. Sometimes the mucous membrane becomes studded with lardaceous and encephaloid vegetations, while the serous coat presents cartilaginous tubercles.

In two cases, Dr. Bushe has seen the cartilaginous transformation uncombined—in one, the muscular coat and cellular tissue were affected—in the other, vegetations studded the mucous tunic.

The encephaloid transformation may commence primarily in the cellular tissue or mucous membrane, or may follow the cartilaginous or lardaceous changes.

Such is Dr. Bushe's account of these malignant alterations. He candidly admits its insufficiency.

Mr. Syme's remarks are still more general. He simply states that the morbid growth generally possesses a moderate degree of firmness, and exhibits characters intermediate between those of carcinoma and medullary sarcoma. A good account of the malignant alterations of the rectum continues a desideratum.

From these changes of structure, whatever they may be, the cavity of the intestine, as Dr. Bushe remarks, is diminished, but this is not at all in proportion to the amount of disease, for the quantity of carcinomatous matter in any one part may not be great, though several inches of the intestine may be diseased. Indeed, it frequently happens that, though the bowel may have been considerably obstructed for some time, the softening down and separation of the projecting masses, again renders it pervious.

Mr. Syme observes that there has been some difference of opinion as to the comparative frequency of simple and malignant stricture of the rectum. But, from his own observation, he thinks that the latter is more often met with than the former.

Any portion of the rectum may be first attacked; the disease most commonly, however, commences at its junction with the sigmoid flexure of the colon; then, immediately above the pouch, and lastly at the anus.

Mr. Syme states that it generally occurs in the same part of the gut as the simple stricture, but is not so limited or regular in its extent. The diseased growth is sometimes confined to one side of the gut, at others it affects the whole circumference; and it is only in the latter case that there is stricture properly speaking. The swelling is usually of a very irregular form, and seldom extends less than several inches along the gut. Occasionally it descends quite to the anus, or even shows itself externally, but more frequently it leaves the coats of the intestine free for an inch or two within the sphincter.

The adjacent organs, as the bladder and vagina, are most commonly involved.

The causes of the disease are involved in great obscurity.

Those about or a little above the meridian of life, are most liable to this disease. No age, however, is exempt from it. Dr. Bushe has seen the encephaloid transformation in a boy twelve years old, and the lardaceous in a female of twenty-three.

From an examination of published cases, Dr. Bushe finds that women, and more particularly those who have recently ceased to menstruate, are most commonly victims to the malady.

When the finger is introduced, says Dr. Bushe, we may discover, firstly, that the intestine is firmer than usual, and a portion of its inner surface is covered with indurated tubercles; secondly, that it is hard and contracted for a considerable extent, and the mucous membrane studded with ulcers, more or less extensive, whose surface is unequal, granular or fungous, and surmounted with thick, hard, and everted edges; thirdly, that a firm cartilaginous ring, generally with an uneven surface, and so extensive as barely to admit the extremity of the finger, occupies its circumference; fourthly, that a portion of its inner surface is rendered irregular, and its cavity lessened by soft fungous growths; and fifthly, that the disease is confined to its lower extremity, and a fungus is thrown out either from a part, or all the circumference of the anus.

The following is Dr. Bushe's account of the symptoms usually displayed by a patient:—

"He suffers a burning pain in the rectum, which shoots through the pelvis. He is also tormented with weight in the back, aching above the pubes, numbness of the thighs, and painful retraction of the anus. His stools are frequent, difficult, painful, scanty, slimy, dark-coloured, and mixed with blood and matter of an ichorous quality. In some instances, however, they are figured or composed of small pellets, and occasionally they are liquid, abundant, and accompanied with dreadful tenesmus. He, moreover, labours under abdominal pain and distension, eructions, hiccough, nausea, and severe vesical irritation. Frequently, he cannot sit, and in some instances is unable to walk, only obtaining relief in the recumbent position. He loses his flesh and strength; the ichorous discharge increases, and runs out when he coughs, or even when he stands erect. There is occasionally considerable hæmorrhage, and he becomes sallow or leaden coloured, œdematous, and sinks under continued suffering. Sometimes however, when the disease is of a fungoid character, he may die from obstruction.

The excessive shooting pain through the pelvis, the sallow or leaden colour of the face, and the havoc made by the disease in the advanced state, enables us to distinguish it from stricture. It must be confessed, however, that unless it commences in the form of indurated tubercles or irregular fungoid growths, the diagnosis is not easy in the commencement." 293.

*Treatment.*—On this it is, unfortunately, idle to dilate. The relief of pain by sedatives and by the hip-bath—the gentlest aperients, as castor-oil, or mild enemata—the horizontal posture—and light nutritious diet, are the chief general remedies that experience can approve of. Bougies can do no good, and may do much harm.

The following are the sentiments of Dr. Bushe on the subject of removing the diseased portion of the bowel:—

"When the disease is confined to the extremity of the bowel, and does not extend upwards beyond two and a half or three inches, provided the general health be good, we may, if the patient desires it, remove the affected parts, though, I would much rather avoid any such proceeding; for the return of the disease will be more than probable. Four years ago, I performed this operation on a man, in whom the cancerous transformation seemed confined to an inch and a half of the intestine, and in all probability commenced at the verge of the anus. I made an elliptical incision around the anus, with the extremities pointing, one forwards, and the other backwards, and by prosecuting the dissection inwards and upwards removed, without the least difficulty, the diseased extremity of the bowel. I found Museux's forceps very useful in bearing off the intestine during the dissection, and as I thought it all-important to see my way clearly, I secured the arteries, six or eight in number, as they were divided. The wound healed rapidly, and his general health improved; but there was a slight prolapsus of the bowel, which, however, was completely supported by prepared sponge, covered with oiled silk, and an elastic bandage. When the feces were liquid, he could not retain them; but when solid, he was generally able to anticipate their discharge, very little being expelled at each time. This man continued well for a few months, and returned to the country, but died, as I have been informed by his wife, of what she called consumption, seven months after the operation."* 295.

Mr. Syme remarks that, it appears that a considerable portion of the rectum, even to the extent of a couple of inches, may be cut out without immediately fatal or any very serious bad consequences in the first instance. But the patient can experience no benefit from this being done, and, in addition to the pain of the operation, must have an impulse given to the morbid action. And if there are any cases in which this excision of the rectum has been followed by a permanent cure, the disease could not have been of a malignant nature.

We agree with Mr. Syme. If the disease is really malignant the operation will be, in all human probability, useless. If it is not malignant, the operation is not only unnecessary but cruel. Bad as are the chances from operations for malignant diseases every where, they are peculiarly bad about the rectum, and we think that the surgeon who removes the lower end of the bowel in cases of this description, is probably conferring no benefit on either science or his patient.

But what shall we say to the following startling statement made by Mr. Syme?

"It may seem unlikely that so severe a proceeding should ever be resorted to except in cases the most hopelessly incurable by other means. But, so far from this, however startling and incredible it may appear, the fact is, that removal of the extremity of the rectum has of late years been taught and practised in this city, as the best mode of treating those hemorrhoidal affections which are generally comprehended under the title of *prolapsus ani*. That a complaint which, as has been shown above, may be certainly remedied with little pain, no danger, and without any injury to the natural structure, should give occasion to an operation so dreadful in its performance and effects, as cutting out the end of the bowel, together with its sphincter, is to be deeply regretted, as well for the credit of surgery as the good of humanity. It is needless to say that, after this extirpation has been performed, the healing of the wound is attended with an extreme contraction, I have heard even obliteration of the gut; and the patient must consequently, like the victim

* Lisfranc was the first who performed this operation: he has repeated it many times, as well as other surgeons.

of ancient operation for fistula, suffer from the united miseries of constipation and incontinence." 131.

It is needless to expend words on the reprobation of such extravagantly bad practice.

Mr. Syme adds that, it is possible that cancer may occur at the verge of the anus, as it does in the somewhat similar texture of the lip, and then excision may be practised without any impropriety. But cases of this kind are extremely rare, and should be carefully distinguished from those in which the coats of the bowel are implicated, where the knife can never be prudently or beneficially applied.

### D. Polypus of the Rectum.

The last of the diseases of the rectum to which we shall direct attention, is Polypus. It is described in the fourth chapter of Mr. Syme's work, and the twenty-first of Dr. Bushe's.

Polypi occasionally form in the rectum. Sir A Cooper says, that, in the whole course of his experience, he has met with only ten cases of this description.

Sir A. Cooper says also, that the disease generally occurs in children, and very rarely in adults. The most advanced age at which he has met with it was twenty-two. In the few cases that have occurred to Mr. Syme, the patients had attained or passed the middle period of life. Dr. Bushe observes that, if we may form any opinion from the cases of polypi of the rectum that have been recorded, they appear to have generally occurred in adults, and for the most part in females.

The causes of the malady are involved in total obscurity. To say that they result from the operation of circumstances of an irritating nature is to seem to say much, but really to say little.

The nature and forms of the tumor can scarcely be considered very determinate. They are generally, says Dr. Bushe, of the mucous, though sometimes of the sarcomatous species. They may be multiplied or solitary. In the majority of cases, they are situated near the anus, though they are not unfrequently beyond the reach of the finger. They are generally globular, being pedunculated or sessile. Oftentimes, they seem to be made up of the reunion of many lobes. Mucous polypi are developed very slowly, and never grow to any great size, varying from that of a pea, to that of a pullet's egg. Sarcomatous polypi, on the contrary, grow rapidly, and attain a very considerable magnitude. Mr. Syme has always found Polypus of the Rectum single. The tumor, according to him, is of a round or pear-shape, varying in size from that of a pea to the bulk of a hen's egg, and either smooth or lobulated on the surface. It has a narrow neck or footstalk, which is usually attached within an inch or two of the anus. In its consistence there is considerable variety, the texture being sometimes firm and unyielding, at other times soft, and hardly distinguishable by touch or ocular inspection from the lining coat of the bowel.

Dr. Bushe gives a fuller account of the symptoms, than that presented by Mr. Syme :—

"Those afflicted with this malady, complain of weight and fullness in the rectum, tenesmus, and difficulty in defecation. The evacuations, when soft, are contracted, flattened, and generally besmeared with blood, mucus, or pus, so as to lead to the belief, that there is stricture of the rectum, but the touch at once determines the point. If the polypus be situated near the anus, it will descend during stool, and when large, can only be returned with difficulty. In some rare instances, the bowel contracts with so much force, as to detach the tumour. The late Mr. ——, on whom I attended in consultation with Dr. Stevenson, discharged a large mucous polypus in this way.

Mucous polypi are not very sensible, nor are they dangerous, if within reach and attended to in time; but should they be neglected, they may degenerate, and prove fatal. The fungous polypi are, however, much more sensible, and as they are prone to ulceration, the result will generally be fatal, because of the almost impossibility of removing every part of them, and the certain return of the disease, if this be not effected.

When the polypi increase in size and malignancy, the patient becomes sallow, and loses his appetite; his tongue is coated, and his thirst intense. He is troubled with flatulence, and colic pains. Emaciation, œdema, and hectic fever now set in. The fecal discharges can only be effected with difficulty, and in small quantity, and even this cannot be accomplished without the aid of enemata or medicine. The tenesmus and weight in the rectum increases; there is much muco-purulent discharge, lancinating pains, and, frequently, considerable hemorrhage." 229.

*Treatment.* If the neighbouring parts are sound, these polypi should be, by all means, removed. Dr. Bushe recommends that tepid-water should be injected into the rectum, and when, by its evacuation, the tumor is prolapsed, the patient ought to lie on his side, and while an assistant separates the buttocks, the surgeon should seize the polypus with Museux's forceps, and cut it away with a curved scissors. If there be a plurality of these growths, they ought to be treated in the same manner. In some instances, the polypi do not descend with the evacuation of the water, and then we shall be compelled to dilate the anus with the speculum, and having carefully secured the tumor, and nothing else, in the forceps, to drag it down. Dr. Bushe has twice done this without hæmorrhage. Dr. Bushe alludes to the ligature, but neither praises nor dispraises it.

Mr. Syme prefers the ligature on account of the possibility of hæmorrhage.

"In a case which I saw with Dr. Hilson of Jedburgh, the tumour, which was about the size of a cherry, and appeared to have existed for upwards of twenty years, was attached to the posterior surface of the gut by a slender footstalk, long enough to permit its being readily protruded from the anus. A single ligature might have proved effectual, but, to make the strangulation as complete as possible, I passed a needle through the root of the growth, and tied each of its halves with a separate thread. The patient, who was a lady upwards of seventy years of age, recovered without any inconvenience. In another case, for which I am indebted to Mr. Craig of Ratho, it was necessary to pursue a different method.

'In January 1835, Mrs. H. aged 44, was delivered of her ninth child. The labour was in every respect natural and easy, and she made a good recovery.

On the 2nd of April 1836, I was sent for to visit her, and upon my arrival found that she had had a very profuse discharge of blood from the rectum. She was pale and exhausted, with a small feeble pulse. As the bleeding had ceased, and I was unwilling to disturb her, I merely prescribed the horizontal posture and doses of acetate of lead, with Dover's powder every four or five hours. The quantity of blood discharged could not be accurately ascertained. I saw two common water-pots nearly full, the one with scybalous feces and blood, the other apparently with blood. But she told me that more had been previously passed to the extent of inducing syncope. There was no return of the bleeding, and she made a good recovery.

On investigating the nature of this case, I was informed by the patient that for fifteen years she had been more or less annoyed by uncomfortable sensations in the pelvis, with pain in her back, loins, and thighs: That some years ago, while pregnant, and within six weeks of delivery, she had had a similar bleeding nearly as profuse, after which she made a very tedious recovery: That during the births of her younger children, she had been sensible of an uneasiness in the posterior parts which was not formerly remarked: That when she strained at stool something frequently came down, which required to be returned; and that she had consulted a variety of practitioners for the complaint, without obtaining any relief.

I could hardly think, as seemed to have been previously supposed, that the uterus was the seat of the disease, because her labour had been natural; because the menstrual evacuations were regular; and because when examined it was felt of the ordinary size as well as consistence. Suspecting that piles might be the cause of her complaint, I carefully examined the rectum, and in the hollow of the sacrum detected a large pendulous tumour attached to the gut by a narrow neck. It was lobulated on the surface, and in consistence resembled the placenta or lung. It could not be protruded. But this seemed owing rather to its size than the resistance of its root. Pulling it gently occasioned pain shooting through the pelvis. It was sensible to pressure near its origin, but not in its mass generally. Having no doubt that this polypus was the cause of all her suffering, I proposed its removal; and on the 18th of November Mr. Syme effected this by applying a ligature round it neck. In the course of a few days afterwards the tumour sloughed away in different pieces, which did not permit its structure to be satisfactorily ascertained. After this her general health improved, and she was relieved from all the disagreeable sensations which had so long distressed her, and at length produced a serious depression of spirits.'

Great credit is due to Mr. Craig for discovering the polypus in this case, since the consistence of the tumour, as recognized by touch, was so similar to that of the intestinal coats, that I could not satisfy myself of its presence, except by feeling the neck and tracing it into the body of the swelling. The ligature was passed by introducing it on the point of the finger, carrying it round the footstalk, and then withdrawing it by means of a hook." 107.

When the polypus is very high (we return to Dr. Bushe) it will be impossible to prolapse it. Under such circumstances, the operator may be able to conduct a probe-pointed curved scissors along the finger, and cut through its peduncle, or it may be possible to tie it after the manner of a uterine polypus, as did Desault, even though it was six inches from the anus. Dr. Bushe, however, admits, that, at such a height, there is much danger of including other parts besides the neck of the polypus.

We have now concluded the examination of the various diseases of the rectum. We would fain believe that the three copious articles we have devoted to their consideration will not prove altogether useless. It would be idle to dwell on the necessity for being thoroughly acquainted with these maladies. Frequently distressing, often remediable, their nature weighs heavily on a patient's comfort and repose of mind, and he anxiously seeks relief at the hands of science. Too often he does not receive it.

The work of Dr. Bushe is carelessly written, but certainly the most valuable that has ever appeared upon the subject. Mr. Syme's book is concise, clear, and practical. It does not aim at the comprehensiveness of Dr. Bushe's, but so far as it goes, it is likely to prove a valuable addition to the library of an operating surgeon.

